# Persistence Increases with Diversity and Connectance in Trophic Metacommunities

**DOI:** 10.1371/journal.pone.0019374

**Published:** 2011-05-27

**Authors:** Dominique Gravel, Elsa Canard, Frédéric Guichard, Nicolas Mouquet

**Affiliations:** 1 Department of Biology, McGill University, Montréal, Quebec, Canada; 2 Centre d'étude de la forêt and Département de biologie, chimie et géographie, Université du Québec à Rimouski, Rimouski, Québec, Canada; 3 Institut des Sciences de l'Evolution, Université Montpellier 2, Montpellier, France; 4 Department of Biology, McGill University, Montréal, Québec, Canada; 5 Institut des Sciences de l'Evolution, Université Montpellier 2, Montpellier, France; University of Zurich, Switzerland

## Abstract

**Background:**

We are interested in understanding if metacommunity dynamics contribute to the persistence of complex spatial food webs subject to colonization-extinction dynamics. We study persistence as a measure of stability of communities within discrete patches, and ask how do species diversity, connectance, and topology influence it in spatially structured food webs.

**Methodology/Principal Findings:**

We answer this question first by identifying two general mechanisms linking topology of simple food web modules and persistence at the regional scale. We then assess the robustness of these mechanisms to more complex food webs with simulations based on randomly created and empirical webs found in the literature. We find that linkage proximity to primary producers and food web diversity generate a positive relationship between complexity and persistence in spatial food webs. The comparison between empirical and randomly created food webs reveal that the most important element for food web persistence under spatial colonization-extinction dynamics is the degree distribution: the number of prey species per consumer is more important than their identity.

**Conclusions/Significance:**

With a simple set of rules governing patch colonization and extinction, we have predicted that diversity and connectance promote persistence at the regional scale. The strength of our approach is that it reconciles the effect of complexity on stability at the local and the regional scale. Even if complex food webs are locally prone to extinction, we have shown their complexity could also promote their persistence through regional dynamics. The framework we presented here offers a novel and simple approach to understand the complexity of spatial food webs.

## Introduction

The relationship between food web complexity and stability is amongst the most studied and debated questions in ecology (see reviews [1]–[4]). This question has a long history [5]–[8], vigorously initiated by May [9], followed by extensive modeling studies (e.g. [10]–[16]), and yet to be resolved (e.g. [17]–[21]). The complexity-stability debate has been more recently translated into space for simple webs, where limited dispersal affects the stability of enemy-victim interactions. Numerous studies on dynamical stability have been conducted for simple spatial food webs (e.g. [22]–[23]), with various dispersal functions (e.g. [24]), and in some cases, including spatiotemporal heterogeneity in the environment [25]. There is also a considerable body of work on the spatial stability of simple predator-prey modules (e.g. [26]–[30]). Overall, these studies show that dispersal has the potential to stabilize food web interactions through various mechanisms (see reviews [31]–[34]). However, all of these studies are restricted to rather small food web modules with few species, and we could hardly extrapolate them to the more complex web configurations found in nature [35]. Consequently, it is crucial to understand whether or not persistence in complex spatial food webs follows the insights gained from the study of simple modules in isolation [36].

Here, we report a study extending previous work on spatial food web ecology [33] by considering more complex and natural food webs. We study persistence as a measure of stability for food webs subjected to patch dynamics and ask how do diversity, connectance, and topology of spatially structured food webs influence it. We study these questions first by analyzing two mechanisms drawn from simple food web modules. Under patch dynamics, it was established for linear chains that species are inevitably *spatially inefficient* at occupying the landscape [37]. We find that in more complex webs, this spatial inefficiency would result in lower persistence of the highest trophic-ranked species. We also find that for more complex structures, persistence increases with the *linkage density* (number of links per consumer) and the *diversity* of primary producer species. Based on these arguments, we hypothesize that complex and diverse food webs will be more persistent than simple ones. We assess this prediction through simulation of artificial and empirical webs, of both variable complexity and diversity. We find a positive relationship between regional food web complexity and persistence and that the distribution of the number of prey per predator is a crucial topological attribute for persistence. Spatial food web ecology is difficult to tackle because of the inherent complexity of food webs [35], scales [38], and frequent idiosyncratic model predictions [33]. Our study contributes to the solution of this problem with the proposition of simple general mechanisms that could accommodate realistic food web topologies and spatial configurations.

## Analysis

### Defining stability in trophic metacommunities

Stability has numerous mathematical definitions [1], [13], [39]–[40], but these definitions share common ground in that a system is called “stable” when it returns to equilibrium following a disturbance [9]. First analytical work was based on local stability analysis [9], but recent studies have shifted the focus to a global metric of stability based on persistence [18], [35], [41]–[43]. Here, we start from the assumption that a given food web is feasible at the local scale (with no consideration of its dynamical stability sensu May [9]) and that the local population extinction from environmental stochasticy (patch dynamics) is interpreted as the disturbance affecting this food web. Persistence encompasses both local stability and attractor trajectories [13]. In this context, we adopt persistence as our metric of stability, defined as the fraction of remaining species at equilibrium of a food web subject to colonization-extinction dynamics (as in refs 18,35,41–43). Although it does not allow a strict comparison with more traditional metrics of dynamical stability, persistence is a more appropriate metric for trophic metacommunities with patch dynamics.

### A colonization-extinction model of trophic metacommunity

The fundamental principle underlying most metapopulation ecology is that the dynamics of an ensemble of local populations can be described as a balance between colonization of empty patches from occupied ones and extinction of occupied patches resulting from environmental and demographic stochasticity. Here, with the help of simple food web modules [36], [44], we use a model to interpret general mechanisms affecting persistence of spatial food webs with no limit to their complexity. The model assumes an infinite and homogeneous landscape divided into patches, global dispersal, and fast local dynamics relative to regional dynamics [45]. Occupied patches are of identical quality, regardless of the number of resident prey species, and thus, the colonization rate is proportional to the fraction of occupied patches in the landscape. Following Holt [37], we note the fraction of landscape suitable for colonization (*h_i_*) by a predator species *i* is the fraction of all patches in the landscape occupied by at least one of its prey species. All space is available (*h* = 1) to primary producers. Our results hold qualitatively under more stringent situations where a consumer needs more than one prey to survive (results not shown). We also assume that more than one predator can occupy a patch occupied by a given prey species. The following equation describes occupancy dynamics for species *i*:
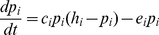
(1)where *p_i_* is the fraction of landscape occupied by species *i*, *c_i_* is the colonization rate, *h_i_* is the fraction of the landscape suitable for species *i*, and *e_i_* is the extinction rate. For tractability, this model assumes donor-controlled metapopulation dynamics, because the extinction rate of a prey in Eq. 1 is independent of the presence of its predators in the patch. We however relaxed this assumption with simulations (see below) and found that our results are robust to recipient-controlled dynamics. The essential result of this model is that species *i* persists at equilibrium provided that h_i_>*e_i_*/*c_i_*; the fraction of suitable patches (i.e. occupied by prey) is an important property and must be large enough to withstand colonization-extinction dynamics [45]. We now examine how topological attributes of food webs affect this prediction through two mechanisms: spatial inefficiency and linkage proximity to primary producers.

### Two fundamental mechanisms for regional persistence

#### Mechanism 1: The fundamental constraint of spatial inefficiency

**Figure 1:**
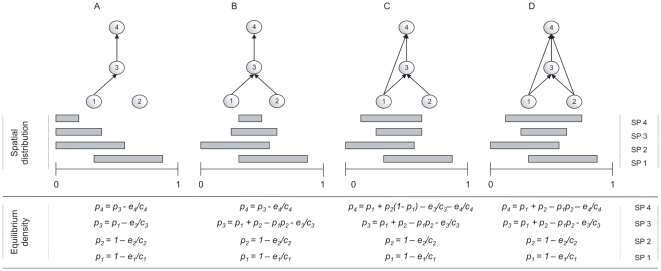
Calculation of available patches for simple food webs. The webs are ordered from left to right by their connectance. The grey bars illustrate the spatial co-distribution of species. The length of the bars depicts the equilibrium spatial occupancy and the superposition with other species in the co-distribution.

At the scale of a local community, there is a longstanding hypothesis that the inefficiency of energy flow through trophic levels limits food chain length [13], [46]–[47]. The amount of suitable patches for establishing a population is a fundamental resource for metapopulations and it is also used inefficiently across trophic levels: because of colonization-extinction dynamics, a consumer could not use all suitable patches (ie. the ones occupied by its preys) and consequently, the fraction of patches that are occupied reduces as we move up in the chain. In mathematical terms, it means the equilibrium density p_i_* of any species subject to colonization-extinction dynamics cannot exceed the quantity of suitable patches *h_i_*. This constraint has dramatic consequences for the persistence of the highest trophic levels [36], [43], [48]. Consider a simple linear food chain of three species (Fig. 1A). The available habitat of a primary producer (*h*_1_ = 1) is greater than that of an herbivore (
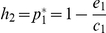
), which in turn exceeds that of a first-level predator (
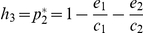
). This solution could easily be extended to the n^th^ level of a longer chain [36], [48] The quantity of available habitat for a species at trophic level *i* in a linear chain is given by:
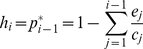
(2)Although extremely simple, this model has clear implications for the persistence of more complex spatial food webs. For an *e/c* ratio independent of trophic rank, the fraction of available patches for colonization decreases linearly with trophic rank. As a consequence, spatial constraints impose a limit on food chain length such that the maximum number of trophic levels 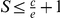. Based upon this result derived by Holt [37], we predict that “long” food webs with a high number of trophic ranks should be less persistent than more “compact” food webs where species are closer to the primary producers.

#### Mechanism 2: Expected linkage proximity to primary producer species

We now extend previous work on linear chains [37], [48] to explore more complex food web structures. Consider a perfect cascade food web [49], with no loops or cannibalism, and where each consumer can feed on all primary producers as well as on all other consumer species of lower rank (e.g. Fig. 1D). We then find the number of patches available for any consumer species will be the total fraction of the landscape occupied by primary producers (assuming each primary producer satisfies the conditions for regional persistence). The performance of primary producers is thus of crucial importance for the higher levels. For a web having two primary producer species (indexed 1 and 2), which are distributed independently over patches, the amount of habitat suitable for colonization by herbivores and predators is 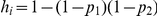 (Fig. 1D). The product 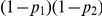 is the fraction of patches that are occupied by neither species 1 nor species 2. The fraction of patches available for consumer species would be independent of the consumer's trophic rank, but strongly dependent on the diversity of primary producers. For this restrictive example of a perfect cascade, we find the fraction of available habitat for a consumer at any trophic rank in a web of *n* primary producers is be given by:
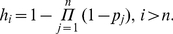
(3)The probability of finding at least one primary producer in a patch increases asymptotically to one with increasing diversity of primary producers (*n*). We note however this finding assumes that primary producers are not interacting with each other and thus encounter each other at random. The result would change quantitatively but not qualitatively if producers are more or less spatially aggregated, for instance because of facilitative or competitive interactions (see *Discussion*). With this simple extension of Holt [37], we predict that, for a perfect cascade, the persistence of a consumer species is a saturating function of the diversity of primary producers and is independent of its trophic rank.

#### Persistence in complex food webs

Real food webs are rarely (or perhaps never) linear chains or perfect cascades. For intermediate cases between these two extremes, the constraint of spatial inefficiency (Mechanism 1) on persistence will differ from one web to another. For instance, we see that the available habitat *h*_4_ for the top species 4 in Fig. 1 increases as it feeds closer to the primary producers (comparing webs B-C-D) and that it could even be equal to or greater than the one of lower-ranked species (e.g. sp. 3 vs. sp. 4 in Fig. 1D). For webs that are intermediate between the perfect cascade and linear chain, we cannot find a general analytical solution for the fraction of available patches. This solution is specific to the web topology and becomes rapidly complex with increasing species diversity because it requires tracking co-distributions between all pairs of prey species. We can however get an intuitive understanding of the effect of diversity on the number of suitable patches based on a simple line of reasoning. In a web with a random structure of *S* species and directed connectance *C* (*C = L/S^2^*), where *L* is the number of feeding links in the web (ranging between 0 and 1), the probability that a consumer *j* feeds on a given species *i* is *C*. Suppose there are *n* potential prey species already present in the patch, what is the probability this consumer finds at least one prey among them? With *n = 1*, this probability is *C*; with *n = 2*, this probability is 1 – (*1-C*)(*1-C*) and so it is 1 – (*1-C*)*^n^* for *n* potential preys. This probability asymptotes to one with increasing diversity and connectance. We thus predict that persistence of a spatial food web should increase with diversity and connectance.

### Simulations of complex food webs

We now explore the relationship between persistence, diversity, and connectance with simulations of more complex and realistic food webs structures. We start with simulations of patch dynamics of artificial food webs to control for species richness and connectance. Then, we simulate the dynamics of empirical food web structures and test the importance of food web topology on persistence.

Our metacommunity model is stochastic and spatially implicit. There is no competition between predators (as discussed in the aforementioned analytical models). The metacommunity comprises 250 patches. At each time step, we successively update all patches that are selected at random to approximate a continuous time process [50]. We now relax the above assumption that a predator has no effect on its prey's extinction rate. The extinction probability of a consumer has a value of 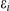 when at least one of its preys is present (excluding cannibalistic links) but not its predator, 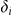 in the presence of its prey and its predator and 1 when no prey is present. The predator-prey interactions are thus recipient-controlled when 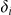 is larger than 0. The probability of a species colonizes a patch it does not occupy is *c_i_p_i_* if at least one of its prey species is present, with *p_i_* being the fraction of the 250 patches it occupies in the landscape, or 0 if it has no prey species present. Simulations start with 100% occupation of patches by all species and are iterated to equilibrium.

We first performed simulations with artificial food webs of *S* species with directed connectance *C*. At the end of each simulation, we recorded the number of species that went extinct from the original food web to calculate persistence (the fraction of species remaining). The first food web structure that we analyze is a random interaction matrix. For each possible link in the *S* ^2^ matrix, the probability of a feeding link was *C* (thus including cannibalism and cycles of up to *S* species). For each random web matrix, we checked for isolated species and loops (i.e. loops that are not connected to a primary producer) and redrew links at random for these species. The number of primary producers was kept constant for all webs to focus on connectance. To analyze the impacts of web structure we then generated artificially structured webs according to the niche model of Williams and Martinez [51]. Again, the number of primary producers is held constant and links are redrawn following the same procedure for isolated species. We also simulated the cascade model [49], but the results were qualitatively similar to those of the niche model, and thus, we only present results for the former. We independently increased connectance to span the range observed with real webs and total diversity from 25 to 150 species (keeping the number of primary producers constant). The role of spatial dynamics was assessed by running simulations across a gradient of colonization rates while controlling for the extinction rate. One hundred replicated simulations were conducted for each combination of parameters (connectance, diversity and colonization rate).

We also simulated the spatial dynamics with real food web structures. We used interaction matrices from 175 published food webs that were compiled by Cohen [49], [52] (107 webs), Havens [53] (50 webs) and Dunne *et al.* [54] (15 webs). The dataset has a wide distribution of diversity (5–181 species) and connectance (0.016–0.33; Table 1 summarizes some essential descriptors of the dataset). Our results are robust to variability in taxonomic resolution across food web resolutions [3], [55]–[56](see below). We randomized the identity of prey or predator species in empirical webs to remove specific topological attributes, such as the degree distribution (number of trophic links per species) and distance to primary producers, while conserving others. A food web matrix of size *S* ^2^ is organized with predators as columns and prey as rows. In the first scenario, we altered the degree distribution by randomizing of the links within rows, so that the identity of predators for a prey changes but their number remains constant (permutation of predators). This is a severe change in the food web structure, as it considerably changes the distribution of the number of preys per predator (i.e. the degree distribution tends to a normal distribution with a much narrower variance). In the second scenario, we kept the number of prey per predator but we changed their identity, by randomizing the links within columns (permutation of prey). Note that for both scenarios, we retained the identity of primary producers so that their diversity was held constant for all randomizations. As was the case with the artificial webs, we checked for isolated species and reshuffled columns or rows when necessary to avoid extinctions. We performed 100 randomizations for each scenario.

**Table 1:** Summary statistics (mean ± SE) for the empirical (n = 175) and permutated webs.

Statistic	Empirical webs	Permutation of predators	Permutation of prey
S	28.9±1.9	Identical to empirical webs	
L	125.2±19.8	Identical to empirical webs	
C	0.12±0	Identical to empirical webs	
% Producers	27.9±1.25	Identical to empirical webs	
% Intermediate	52.9±1.17	53.3±1.15	64.0±1.12
% Top	19.1±1.21	18.4±1.15	7.66±0.54
% Omnivores	35.3±1.67	74.9±1.69	74.8±1.39
Mean number of prey species per consumer	4.48±0.28	4.53±0.28	4.53±0.28
SD number of prey species per consumer	3.75±0.28	1.57±0.06	3.78±0.28
Mean number of consumers species per prey	2.94±0.18	2.97±0.06	2.97±0.06
SD number of consumers species per prey	2.47±0.13	2.45±0.13	1.39±0.03
Mean distance to primary producer (nb. links)	1.34±0.02	1.32±0.02	1.76±0.04
Max distance to primary producer (nb. links)	2.13±0.04	2.18±0.07	3.29±0.09

Each empirical web is permutated 100 times.

### Simulation results

**Figure 2:**
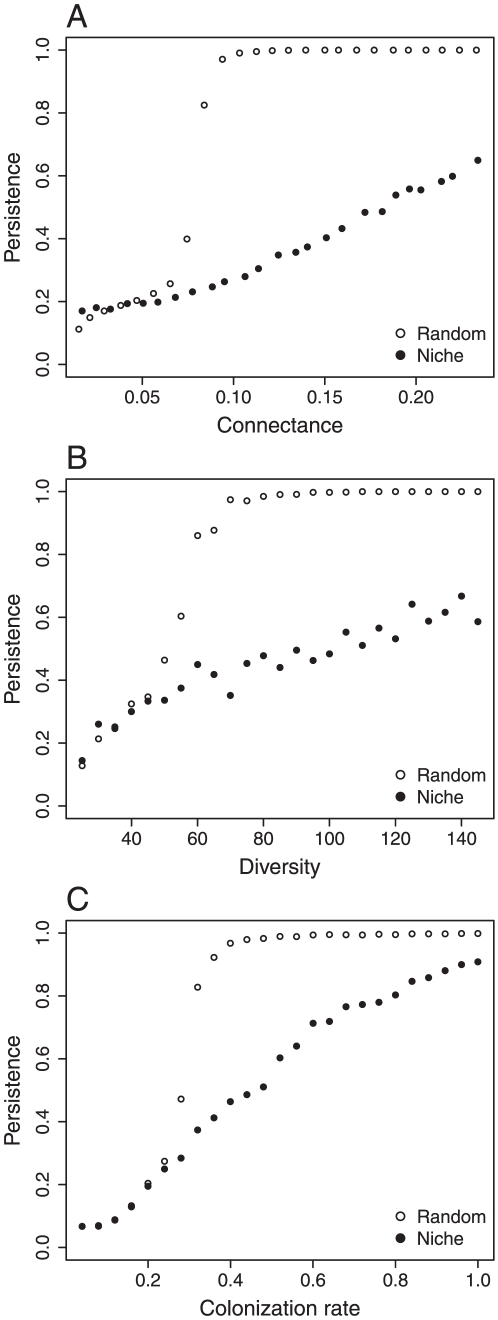
Effects of A) connectance, B) diversity, and C) colonization rate on persistence for webs generated with the niche model and random structures. Each point represents 100 replicated simulations. Parameters are: A) *c* = 0.2, 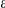 = 0.05, 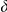 = 0.05, total diversity = 75 species with 5 primary producers; B) *c* = 0.2, 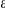 = 0.05, 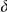 = 0.05, connectance = 0.1 and the proportion of primary producers over total diversity is 0.2; C) 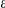 = 0.05, 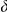 = 0.05, total diversity = 75 species with 5 primary producers and connectance = 0.01.

The simulations with complex webs agree with our expectations derived from simple modules. We first find persistence increases with connectance, diversity, and colonization rate for both random and niche artificial webs (Fig. 2). There is, however, a critical difference in the shape of the relationship between the niche and the random food webs, for all three independent variables. While the niche model has a smooth increase in persistence with connectance, diversity, and colonization rate, the relationship changes abruptly for the random case. There is a threshold characterizing a sharp transition from low to elevated persistence.

**Figure 3:**
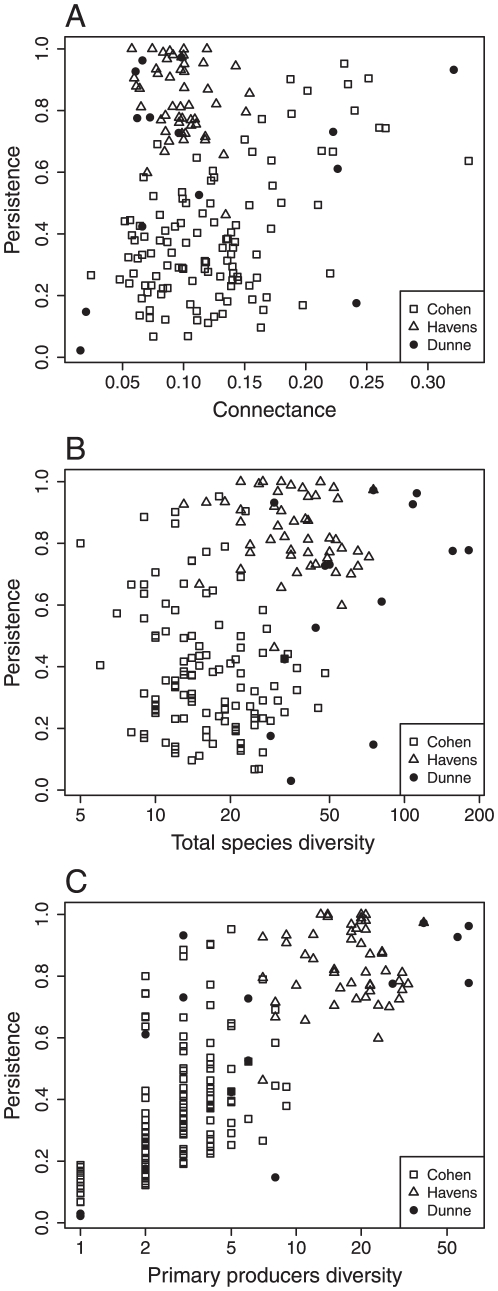
The relationship between A) Connectance, B) total species diversity, C) primary producer diversity and persistence for empirical webs. Each point represents 100 replicated simulations with colonization rate *c* = 0.2 and extinction rates 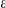 = 0.05 and 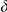 = 0.05. The empirical webs are distinguished for the studies in which they were compiled: Cohen = ref. 49 Dunne = ref. 54 Havens = ref. 53.

**Figure 4:**
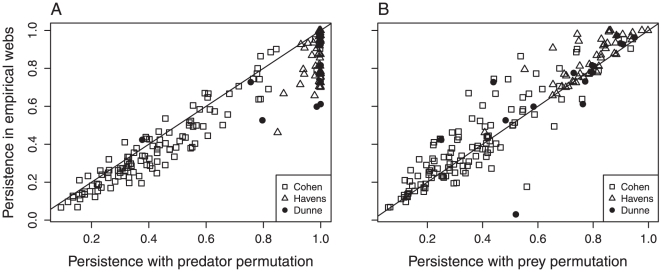
Comparison of persistence between randomized and empirical webs. Parameters are the same as in Fig. 3. A) Within-row randomization (predators); B) Within-column randomization (prey). The straight line represents a 1∶1 relationship.

We tested if real food webs have unique topological attributes affecting their persistence. The results of the simulations based on the attributes of the 175 empirical food webs are presented in Fig. 3. Persistence increases with total species diversity, and primary producer diversity in the empirical webs (Fig. 3A–C). There is however no relationship with connectance because of strong variability in the proportion of primary producers found among the 175 webs (Fig. 3A). The comparison of persistence of the empirical webs with their randomized counterparts (Fig. 4) and reveals that, indeed, real food webs have unique topological attributes affecting their persistence. Randomization of the empirical webs considerably changes the topological properties of the webs, while keeping diversity and complexity constant (Table 1). Randomization increases omnivory for both randomization procedures. Randomization of predators (within-row permutations) reduces variability in the number of preys per consumer: randomized webs have lower numbers of specialists (few links per consumer) and super-generalists (elevated number of links per consumer). As a consequence, randomization of predators creates webs that are more persistent than their empirical counterparts, especially at high levels of persistence (Fig. 4A). Randomization of preys (within-column permutations) reduces the proportion of top species and the variability in the number of predators, while it increases both the proportion of intermediate species, the mean and maximal distance to primary producers. Consequently, empirical webs with randomized preys have lower persistence than their unperturbed counterpart (Fig. 4B). This result differs in donor-control regional dynamics, where we find that randomization of preys have no effect on persistence (not shown). These results imply that prey identity and the average distance to primary producers have no effect on persistence and consequently, that the degree distribution has a much stronger effect on persistence than the average distance to primary producers.

## Discussion

The complexity-stability debate was first addressed as a non-spatial problem, using the tools of local stability analysis [9]. Attempts to scale up predictions to spatial dynamics were limited to small food-webs [37], [44]. This study provides the basis for a spatial theory of complex food web persistence through the extension of a classic colonization-extinction metapopulation model.

### Food web stability: the metacommunity perspective

What insights do we gain from a spatial theory of food web stability? We detailed two mechanisms and key topological attributes affecting the persistence of spatially structured food webs. We first presented how the inefficient transfer of the spatial resource between trophic levels [37], [48] impacts the persistence of food webs with various food chain lengths. This mechanism is somewhat analogous to the hypothesized stability constraint on food chain length [11], [13]: it tends to reduce persistence of the least connected food webs, which are those with the highest likelihood of linear chains. We have then shown that connectance and diversity promote persistence because they preclude the negative effect of spatial inefficiency. Network studies in food webs have shown that 95% of species are within three links of one another. This distance further decreases with increasing connectance and diversity, leading to so-called “small world networks” [57]. This tight connection between species in complex webs could favor the spread of perturbations in local food webs [58], but our results suggests that it contributes to their regional persistence in trophic metacommunities.

We considered persistence to quantify stability as a more appropriate metric for a system characterized by colonization-extinction dynamics. We have found a positive complexity-persistence relationship, in contrast to the negative relationship that has been typically studied [1]. Although persistence does not directly compare to traditional approaches based on local stability analysis (e.g. ref [9]), our results are consistent with some recent studies looking at stability from other perspectives. For instance, species-level analysis revealed that small press disturbances have much less impacts on the whole community when applied to generalist species because they propagate much more diffusely [58]. Species deletion simulations also revealed that generalist species are somewhat keystone species, holding the food web together in the face of extinctions [42]. A positive complexity-stability relationship has also been found at the local scale with realistic foraging dynamics [18], [59]. These results together are giving more credit to the early intuition of MacArthur and others [5]–[7] about the positive effect of complexity on stability.

An interesting paradox arises from our results. Food web complexity might be the driver of instability at the local scale, but on the other hand it is at the same time the factor rescuing food webs at the regional scale. The local instability of complex food webs could be considered as one of the driver of patch dynamics, by causing local extinctions. But we have shown that complexity is a factor promoting the persistence of the food web at the regional scale once it is subjected to colonization-extinction dynamics. This situation is akin to the fugitive dynamics promoting the regional persistence of locally unstable predator-prey interactions [60]). Future work should look at the emerging food web structure from this interesting interplay between dynamical stability and persistence.

### Impacts of food web topology

May's analysis has been strongly criticized for ignoring realistic food web structures (e.g. [10], [12]–[13]). Since then, food web theory has been refined to explain the emergence of food web structure and the existence (or lack thereof) of universal scaling of food web topologies [49], [51], [61]. Comparative analyses, such as the one presented in this study, contribute to understand how topological properties of ecological networks influence their stability [43], [62], [63]. Indeed, May noted in the preface to the 2001 edition of his book that “the reorientation of this question [the complexity-stability relationship] to what kinds of connectance patterns are likely to be most resistant to specific kinds of disturbance is of continuing relevance in ecology.” [64]

Contrasting results between random and niche-structured food webs provides the most striking effect of food web topology on persistence. At low values of connectance, low persistence in randomly structured webs is almost independent of connectance until a threshold is reached, above which all species persist. This sharp transition suggests a percolation threshold in random food webs [65]. A percolation threshold occurs in networks when the addition of a few links is sufficient to suddenly make a set of nodes part of the same large network [66]. Networks with connectance above this critical threshold will be robust to extinction, and thus, maintain habitat availability to species following the random deletion of a few species from individual patches. Structured food webs are not prone to this sudden transition in persistence. The niche model is based on a strong hierarchical structure promoting the transfer of resources from the base to the top. Because of this structure, if a preferential resource transfer channel is missing, the chances of an alternative efficient channel are much higher than in a random food web. However, there is a cost to high connectance in structured compared to random food webs, as the hierarchical structure will bring the top species farther away from the basal species than a random species assembly of equivalent connectance. This constraint will make them more prone to extinction.

We also compared the persistence between empirical food webs and their partially randomized counterparts to investigate how topological properties influence persistence. We altered basic topological properties, such as the distribution of the number of prey species per predator, the number of predators per prey, omnivory, distance to basal species, and food chain length. The scenario with randomization of the predators (within-row randomization) increased persistence relative to empirical food webs, while randomization of the preys (within-column randomization) had the opposite effect. The major difference between empirical webs and their randomized counterpart is the alteration of the degree distribution. The most specialized and vulnerable species are more prone to regional extinction [67]. Natural food webs are often characterized by many weakly connected and few highly connected species [54], so dominated by specialized species with few predators. The randomization procedure normalizes this distribution, impacting the prevalence of these species, and thus persistence.

### Refinements of spatial dynamics

The heterogeneity of spatial dynamics was minimized to keep the analysis tractable. We have not considered for instance the influence of species-specific colonization rates. Our analysis of the simple trophic modules however provides some interesting predictions. For linear chains, Holt [37] predicted that persistence would be promoted if dispersal scales positively with trophic rank. This scaling is expected in many systems where foraging area, home range and dispersal scale are related to body-size [24], [38] and thus to trophic structure [68]. We also found that persistence increases with the generality of the diet in more complex food webs. Persistence is thus promoted by a negative relationship between the generality of the diet and the colonization rate. We also considered a spatially homogeneous landscape with patches of equal sizes and similar connectivity. Adding spatial heterogeneity would definitely generate variability in the local food web structure. The persistence would thus be expected to decrease in smaller and less connected patches, promoting the generalist species and the ones that are closer to primary producers [66].

Our analysis with a spatially implicit model holds under the assumption of random encounter between prey species, i.-e. the co-distribution of pairs of species is given by the product of their occupancy. Other co-distribution patterns would have a considerable impact on persistence, as it influences the total space occupied by two or more prey species. It is well established that spatially explicit dynamics are responsible for the aggregation or repulsion of competiors (e.g. [69]–[70]) and of hosts and parasitoids [26], [71]. For instance in Fig. 1B, the total fraction of suitable habitat for species 3 is given by the summed fractions of occupied habitats of prey species 1 and 2, minus the fraction of the landscape which both species occupy. A wide variety of mechanisms will violate this assumption of random encounter of the prey. Negative co-distribution (repulsion) maximizes the number of suitable patches, while positive co-distribution (attraction) reduces the number of suitable patches. Species co-distribution can have considerable impacts on simple webs [72], but more work is needed to understand he importance of such spatial structures in complex food webs with high diversity and connectance.

### Conclusion

It is a considerable challenge to understand the ecological consequences of spatial dynamics in diverse and connected food webs. Most food webs in nature are entangled networks of direct and indirect interactions and are thus by themselves complex systems to synthesize [73], [74]. The challenge is to provide a theory with a minimal set of assumptions and rich predictions for a variety of systems. With a simple set of rules governing patch colonization and extinction, we have predicted the effects of diversity, connectance, and topology of species interactions on food web persistence. Although persistence differs from dynamical stability, the strength of our approach is that it reconciles the effect of complexity on stability at the local and the regional scale. Even if food webs are locally prone to instability, we have shown their complexity could also promote their rescue through regional dynamics.

Our work has wider implications, as conservation ecology needs a synthetic and predictive theory for food web assembly and collapse [75] in the face of disturbances affecting whole landscapes [76]–[77]. For instance, we currently lack a predictive theory to understand how consumers will track their preys during range shifts [78]. There is a myriad of ecological interactions that are potentially sensitive to global changes. But most of all, the structure of ecological networks will change following emigrations and immigrations. The results we presented here clearly identify diverse and complex ecosystems as the most important properties for their maintenance in face of these changes.
